# Molecular and cytogenetic characterization of *Osteospermum fruticosum* lines harboring wild type pRi *rol* genes

**DOI:** 10.1371/journal.pone.0306905

**Published:** 2024-09-19

**Authors:** Siel Desmet, Katrijn Van Laere, Johan Van Huylenbroeck, Danny Geelen, Ellen De Keyser, Emmy Dhooghe

**Affiliations:** 1 Plant Sciences Unit, Flanders Research Institute for Agriculture, Fisheries and Food (ILVO), Melle, Belgium; 2 Department Plants and Crops, Faculty of Bioscience Engineering, Ghent University, Ghent, Belgium; University of Kotli, PAKISTAN

## Abstract

Transgenic lines engineered through wild type *Rhizobium rhizogenes* display an altered phenotype known as the Ri phenotype. This phenotype includes a more compact plant habit, which has proved useful to obtain more compact varieties that require less chemical growth regulation. Here, we develop a method for the molecular and cytogenetic characterization of Cape daisy (*Osteospermum fruticosum* Norl.) Ri lines in order to predict segregation of pRi T-DNA genes. Analysis of copy number variation (CNV) by means of digital PCR indicated large variation in the copy number of the inserted root oncogenic loci (*rol*) genes, ranging from 1 to more than 15 copies. In addition, up to 9 copies of the auxin biosynthesis genes (*aux*) were present in a single Ri line. Visualization of pRiA4 and pRi1724 *rol* and *aux* insertion in 4 Ri lines was performed through Fluorescence In Situ Hybridization. The number of *rol* integrated loci varied from 1 to 3 loci. In contrast, the different T_R_-gene copies were confined to a single locus which consistently co-localized with a T_L_ locus, this was demonstrated for the first time. Based on CNV and FISH a single Ri line, harboring 7 pRi1724 *rol* gene copies dispersed over 3 integration loci, was selected for breeding. Copy number segregation in R1 progeny of 2, 3, 4 and 5 pRi1724 copies was confirmed, indicating that the evaluation of the breeding value of first generation Ri lines is possible through CNV and FISH.

## Introduction

The root inducing (Ri) plasmid is a unique virulence plasmid responsible for the natural transfer mechanism of rhizogenic agrobacteria [1]. *Rhizobium rhizogenes* is known to carry this Ri plasmid (pRi) and can transfect host plants by inserting the transferrable part (T-DNA) of the plasmid into the host nuclear genome [2]. This horizontal gene transfer event leads to the proliferation of hairy roots (HR) which provide shelter and nutritional benefits for the pathogenic agrobacteria. Research into the biology and genetics of HR enabled the development of HR cultures as a highly technological tool with numerous applications in biotechnology and pharmaceutics [3]. It was observed that axenic HR cultures of several plant species could spontaneously regenerate shoots that, upon transfer to in vivo conditions, displayed peculiar phenotypic traits including, but not limited to, wrinkled leaves, increased branching and a compact, dwarfed plant habit [4, 5]. This phenotype, also known as the Ri phenotype, is the direct consequence of active expression of pRi T-DNA genes that influence plant hormone homoeostasis. The presence of T-DNA-like sequences (cellular T-DNA) in plant genomes has been observed in many species, indicating that in some instances the expression of cellular T-DNA has the potential to impart beneficial effects [6]. Similarly, the Ri phenotype has attracted the interest of plant breeders, which led to the creation of Ri lines for many agricultural and horticultural crops after transformation with wild type rhizogenic strains. To date, the Ri phenotype of more than 80 plant species has been described [7]. Some traits of the Ri phenotype are useful for improving the genetic diversity and have potential to create novel ornamental varieties [8], e.g. more compact varieties that are less dependent on chemical growth regulation, and are more resistant to lodging [9, 10]. However, the implementation of Ri lines in commercial breeding programs is only well documented for several plant species, and only a small fraction of studies report on further breeding with Ri lines [11]. A strategy proposed to facilitate the implementation of Ri breeding on a larger scale is through detailed phenotypic and molecular characterization of Ri lines [5, 7].

Depending on the type of rhizogenic strain used to create Ri lines, different T-DNA integration patterns and combinations can occur. Split T-DNA (agropine) strains, carry a left and right T-DNA fragment (T_L_- and T_R_-DNA resp.) which are independently transferred to the host plant [12]. Oncogenes located on the T_L_-DNA include the *rol* (root oncogenic loci) genes *rolA*, *rolB*, *rolC* and *rolD*, ORF8 and ORF13 [1, 13–15]. The T_R_-DNA of agropine strains also carries two auxin biosynthesis genes *aux1* and *aux2* [16], that share homology with the *tms* genes of the tumor inducing T-DNA [17]. In contrast, the single T-DNA type strains (mannopine, cucumopine and mikimopine) have a solitary T-DNA fragment that harbors the *rol* genes, but does not contain the *aux* genes [18]. Truncated T_L_- or T_R_-DNA integration can also occur, resulting in different combinations of pRi oncogenes being present [19]. Furthermore, different copies of the T-DNA can be inserted at a single locus or at multiple loci in the host plant genome [20]. Digital PCR (dPCR) was proposed as a novel technique enabling the measurement of transgene copy numbers with high precision and relative low labor input compared to conventional techniques such as Southern blot and quantitative polymerase chain reaction (qPCR) [21]. Analysis of copy number variation (CNV) by dPCR was very recently applied in *Sinningia speciosa* Ri lines showing differences in pRi T-DNA copy numbers [22]. Depending on the copy number and degree of dispersion of T-DNA throughout the host plant, homology-dependent gene silencing could occur, resulting in the lack of an apparent Ri phenotype [23]. However, Ri lines without a marked phenotype due to silencing effects, can in theory yield a differential copy number in the offspring if segregation of T-DNA based on multiple loci occurs. Since segregation is likely to occur more frequently in multiple loci, a cytogenetic approach could be implemented to distinguish multiple loci from single locus Ri lines. Fluorescence in situ hybridization (FISH) is a technique that allows the detection of specific DNA sequences on the plant chromosomes by use of fluorescently labeled probes [24]. The physical mapping of the pRi T-DNA on the karyotype of Ri lines could function as the missing link between CNV and predicting the segregation of T-DNA copy number [25].

*Osteospermum fruticosum* Norl. (Cape daisy) is an ornamental perennial plant that has seen a marked increase in popularity in recent years [26]. Cape daisy is generally mass produced vegetatively via cutting and sold as a potted plant. One of the key challenges in producing quality *O*. *fruticosum* is maintaining a compact and sturdy plant habit. Although often achieved via chemical growth regulation, plant growers today are faced with more strict regulation on chemical growth retardants, limiting the options for adequate growth regulation in the near future. In order to tackle this dependence on chemical growth regulation in Cape daisy production, we previously employed a genetic engineering strategy based on wild type *R*. *rhizogenes* [10]. There we have reported on the genotype and phenotype of several unique Ri lines of Cape daisy. These lines exhibited a varying degree of compact growth without the use of chemical growth regulation. In order to promote the use of these Ri lines in future *O*. *fruticosum* breeding and eliminate the need for chemical growth regulation, we set out to provide a set of hands-on tools for the molecular and cytogenetic characterization of these Ri lines, and thus facilitate their use in commercial breeding. CNV was performed by means of dPCR on all lines. Based on CNV, genotype and bacterial strain, four Ri lines were selected for cytogenetic characterization. We created a method to (1) procedurally determine the copy number of inserted T_L_- and T_R_-DNA genes of Ri lines based on CNV, (2) identify Ri lines with separate loci for T_L_- and T_R_-DNA and (3) predict segregation of T-DNA copy numbers in R1 progeny by cross-fertilization of *O*. *fruticosum* Ri lines.

## Materials and methods

### Plant material and acclimatization

An overview of the *O*. *fruticosum* genotypes (wild types o1, o2, o4 and o6) and Ri (Reg1 –Reg10) lines is presented in Desmet et al. [10]. Since then, an additional line (Reg11) was obtained with a similar protocol. Reg11 originates from genotype o1 and was obtained via hairy root regeneration after co-cultivation with *R*. *rhizogenes* strain ATCC15834. Molecular confirmation of the transgenic nature of Ri line Reg11 was done according to the two-step qPCR protocol as described in Desmet et al. [10].

Shoots of all these 11 Ri lines were maintained as axenic shoot cultures. To obtain root tips for preparation of chromosome spreads, micro-cuttings were excised from axenic cultures with 3 internodes and transferred to 150 insert trays filled with a non-fertilized peat-sand (2:1 ratio) mixture and covered with a glass plate (relative humidity > 90%, heating from 15°C and ventilation if > 21°C). After 10 days, the glass plate was gradually removed over the course of 4 days after which the plantlets were grown for another 6 days before being transplanted to individual terracotta pots (Ø 5 cm) and grown in a peat based substrate (1.5 kg.m^-3^ fertilizer: 12N:14P:24K + trace elements, pH 5.0–6.5, EC 450 μS.cm^-1^, Van Israel, Geraardsbergen, Belgium) to stimulate root growth.

### Analysis of copy number variation

RNA extraction, sequencing and library construction were performed as described in Desmet et al. [22]. *Arabidopsis* candidate single copy reference genes proposed by Czechowski et al. [27] were blasted in contigs with minimum length of 400 bp, which resulted in 15 potential reference genes for *O*. *fruticosum*. After validation and gradient analysis (65–56°C) in dPCR, 2 reference genes (*RG3*, *RG7*) were retained as single copy reference genes suitable for quantification of pRi T-DNA genes. Specific dPCR primers are presented in Table 1.

**Table 1:** Primers used for dPCR and FISH probe design.

Plasmid	Gene / Probe	Primer sequence (5’-3’)	Amplicon (bp)	Ta (°C)	Source
pRiA4	*rolA*	F: TCGGAGTATTATCGCTCGTC R: AAAGGAGTGGTGCTCAGATT	127	57	Desmet et al. [22]
	*rolB*	F: GGTAGCTTGCACCTTCTTTC R: TGATATCCCGAGGGCATTTT	103	57	Desmet et al. [22]
	*rolC*	F: CCTCAAATGAGCGTAAACCC R: CCTCCATAGAAGCAGAGCAT	139	57	Desmet et al. [22]
	*rolD*	F: GCAAGGAGAACAAGCATCTC R: CCTCCCGAAATGGAATTGTG	130	57	Desmet et al. [22]
	*aux2*	F: CAGCCGAGACGATTATCAGA R: CTGACCCCTTCATTGTGATG	94	57	This study
	*aux1*	F: GCCATGCAATAGGGAACAGT R: GTGGTGAGTATGCACGATGG	107	57	This study
	*rolB*_*TR*_	F: ATGTCGTGGCTTATGGGTTC R: CCCAATTTCAAATCGAGGAA	94	57	This study
	pRiA4_ T_L_^a^	F: pRiA4_qPCR_*rolA*_F R: pRiA4_qPCR _*rolD*_R	4338	59	Desmet et al. [22]
	pRiA4_T_R_^a^	F: pRiA4_qPCR _*rolB*_*TR*__F R: pRiA4_qPCR _*aux2*_F	4284	58	Desmet et al. [22]
pRi1724	*rolA*	F: TTGATGATCTGAGCGTCACT R: GGTCTGAATATTCCGGTCCA	117	57	This study
	*rolB*	F: CATCACGTTTGGATTGGAGG R: AAAGCCTTCCAGATCAGGTT	139	57	This study
	*rolC*	F: AGGCATCAGGATTCTTTGGA R: AAAGGTCGAAGGTCAGAGAC	141	57	This study
	*rolD*	F: GCCGGGTTTAAGTATCCTCT R: AGATCTCCCTCCAAATGCTC	107	57	This study
	pRi1724_*rol*^a^	F: pRi1724_qPCR _*rolA*_F R: pRi1724_qPCR _*rolD*_R	4086	56	Desmet et al. [10]
-	*RG3 (AT4G05320)* ^b^	F: GGAAGCAGCTTGAAGATGGA R: ACCACGAAGACGAAGGACAA	86	59	This study
-	*RG7 (AT1G13440)* ^b^	F: CGTCGATGTTTCAGTTGTGGA R: CCTCCTTGATAGCAGCCTTG	83	59	This study

^a^ Primers used for FISH.

^b^ The accession number of the reference genes (RG3 and RG7) refers to the *Arabidopsis* reference gene of Czechowski et al. [27].

*EcoRI* and *BfaI* were selected for fractioning the DNA based on the same criteria for the pRiA4 plasmid as described in Desmet et al. [22]. The same enzymes are appropriate for separating potential pRi1724 T-DNA tandem and inverted repeats (S1 Fig). Genome size of *O*. *fruticosum* was determined on all 4 genotypes according to Denaeghel et al. [28] using *Lycopersicon esculentum* ‘Stupické polní tyčkové rané’ (1,96 pg / 2C; Doležel et al. [29]) as an internal standard. An average genome content of 5.72 pg / 2C was calculated. Based on this value, 15 ng of digested DNA (corresponding with approximately 5245 haploid copies) was used as input for the dPCR. In case of high copy number genes, the DNA concentration had to be lowered to 8–10 ng DNA in order to maintain adequate levels of positive droplets (i.e. > 70%). Conditions for dPCR reactions and droplet generation were similar as described in Desmet et al. [22]. Quantasoft version 1.7.4.0917 (BioRad, Temse, Belgium) was used for data analysis. Amplitude and cluster data were exported and used for calculation of concentrations in ddPCRquant (http://www.ddpcrquant.ugent.be/; Trypsteen et al. [30]. The average of the concentration (copies.μL^-1^) of both reference genes was used as normalization factor for calculating the target gene copy number. Copy number calculations were performed according to the method of Collier et al. [21] using R (version 3.6.0) with RStudio GUI (version 1.1.463).

### Cytogenetic characterization of Ri lines

Based on the presence and copy number of specific pRi T-DNA genes, genotype origin of the Ri line and the *R*. *rhizogenes* strain used during co-cultivation, Ri lines Reg3, Reg8, Reg9 and Reg11 were chosen for further cytogenetic characterization. The o2 genotype was included for karyotype analysis.

#### Chromosome spread preparation

Chromosome spreads were prepared from young apical root tips obtained of rooted cuttings according to the “SteamDrop” method [31]. Root tips were pretreated with 0.5% *w/v* colchicine during 3 h at room temperature (RT) and fixated in a 3:1 *v/v* ethanol:acetic acid solution for 45 min at RT. Cell suspensions were made from the fixated root tips after digestion with 0.6% enzyme mixture (0.6% *w/v* cellulase Onozuka RS (Duchefa Biochemie, Haarlem, The Netherlands), 0.6% *w/v* pectolyase Y-23 (Duchefa Biochemie, Haarlem, The Netherlands) and 0.6% *w/v* cytohelicase (Sigma-Aldrich, Missouri, USA)) during 35 min at 37°C. Chromosome spreads were prepared with 1:1 *v/v* ethanol: acetic acid as fixative 1 and 1:2 *v/v* ethanol:acetic acid as fixative 2. Quality of slides was evaluated using phase contrast microscopy (Zeiss AxioImager M2; Carl Zeiss MicroImaging, Belgium), magnification 200x.

#### Karyotype analysis of *O*. *fruticosum* o2

Good quality slides of o2 were stained with DAPI (0.2 μL DAPI + 20 μL Vectashield (Vector Laboratories, Peterborough, United Kingdom)) and analyzed using a fluorescence microscope (Zeiss AxioImager M2, Carl Zeiss MicroImaging, Belgium), equipped with an Axiocam MRm camera and ZEN-software (Carl Zeiss MicroImaging, Belgium), magnification 1000x. Chromosome identification and measurements were done in DRAWID software version 0.26 [32] based on 10 well-spread metaphases. Chromosome classification was done according to Levan et al. [33].

#### Preparation of fluorescent labelled probes

**Fig 1:**
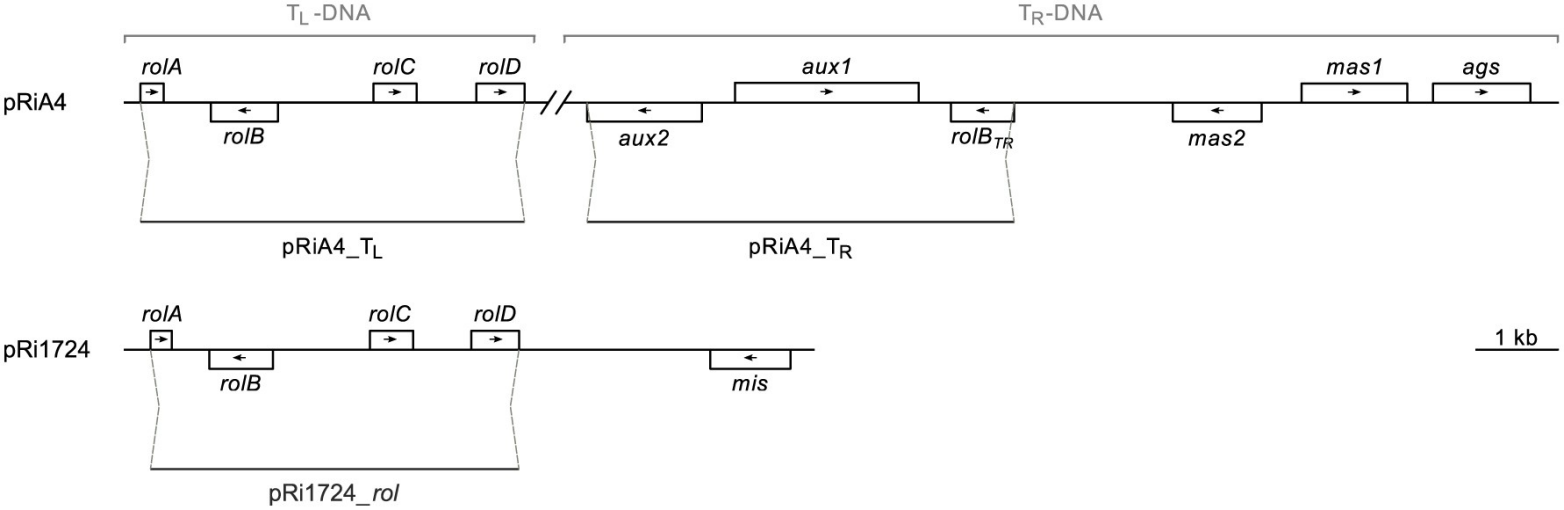
Gene position map of pRiA4 and pRi1724 indicating start and end positions of the different FISH probes. *rolA*–*D* = *root oncogenic loci A–D (arrows indicate ORF direction)*. *aux1–2 = auxin biosynthesis genes 1–2*. *rolB*_*TR*_ = T_R_-DNA homologue of *rolB*. *ags* = *agropine synthase*. *cus* = *cucumopine synthase*. *mas* = *mannopine synthase*. *mis* = *mikimopine synthase*. Scale bar = 1 kb.

Plasmids pTa71 containing the 45S rRNA gene of wheat [34] and pScT7 containing the 5S rRNA gene of rye [35], were labeled by Biotin- (Roche) and Dig-Nick Translation mix (Roche) according to manufacturer’s instructions. For probe design, DNA of strains Arqua1 (plasmid type pRiA4) and MAFF210266 (plasmid type pRi1724) was extracted as described in Desmet et al. [36]. Based on WGS data of the selected strains (available at GenBank as BioProject accession number PRJNA521484 [36]) primer pairs were selected to amplify the T_L_- or T_R_-DNA of pRiA4 (pRiA4_ T_L_ and pRiA4_T_R_ probes) and the T-DNA *rol* genes of pRi1724 (pRi1724_*rol* probe) (Table 1, Fig 1). PCR was performed in 50 μL reactions using 200 ng template DNA, 1 x PCR reaction buffer, 20mM dNTPs, 300 nM of the forward and reverse primers and 3.75 U Expand LongTemplate polymerase (Expand^™^ Long Template PCR System, Roche). PCR conditions consisted of initial denaturation at 94°C for 2 min, followed by 10 cycles of denaturation at 94°C for 10 s, annealing at 60°C for 30 s and extension at 68°C for 3 min, followed by 20 cycles of denaturation at 94°C for 15 s, annealing at 60°C for 30 s and extension at 68°C for 3 min with 20 s extra per cycle and final extension of 7 min at 68°C. The PCR product was purified using the Pure Link Quick Gel Extraction and PCR Purification Combo Kit (Invitrogen) according to manufacturer’s instructions. Purified PCR product was cloned in OmniMAX^™^ 2 T1R chemically competent *Escherichia coli* cell using the TOPO^™^ XL-2 Complete PCR Cloning Kit (Invitrogen) according to manufacturer’s instructions. Successful cloning was evaluated by direct colony PCR of transformed colonies in 25 μL reactions using 1 x GoTaq G2 Flexibuffer, 20 mM dNTPs, 325 mM MgCl_2_, 325 nM of the T3 and T7 primers and 0.6 U GoTaq G2 polymerase (Promega). Direct colony PCR conditions were initial denaturation at 94°C for 10 min, followed by 30 cycles of denaturation at 94°C for 60 s, annealing at 56°C for 60 s, extension at 72°C for 3 min and final extension of 10 min at 72°C. Plasmid DNA was extracted using the QIAprep Spin Miniprep Kit (Qiagen) according to manufacturer’s instructions and consequently labeled by Biotin- (Roche) and Dig-Nick Translation mix (Roche) according to manufacturer’s instructions.

#### FISH and signal analysis

FISH analysis was done on high quality chromosome slides of Reg3, Reg8, Reg9 and Reg11. The protocol described in Schwarzacher et al. [37] was used with the following modifications. Slides were incubated overnight at 37°C and then pretreated with 4% paraformaldehyde in 2x SSC for 8 min at RT and dehydrated in ethanol (70%, 90% and 100%). The hybridization mixture (50% (v/v) deionized formamide, 10% (w/v) dextran sulphate, 2× SSC, 0.25% sodium dodecyl sulphate and 150 ng probe DNA) was denatured at 75°C for 10 min, and then placed on ice for 5 min. On each slide, 80 μL of the denatured hybridization mixture was applied. Slides were then denatured at 80°C for 5 min and incubated overnight at 37°C in a humid chamber. For stringency washing 0.1× SSC was used at 48°C for 30 min. Biotin and digoxigenin labeled probes were detected with streptavidin-Cy3 (Sigma-Aldrich, USA) and anti-dig-FITC (Roche, Germany), respectively. Slides were counterstained with DAPI (0.2 μL DAPI + 20 μL Vectashield (Company)) and analyzed using a fluorescence microscope (Zeiss AxioImager M2 (Carl Zeiss MicroImaging, Belgium)), equipped with an Axiocam MRm camera and ZEN-software (Carl Zeiss MicroImaging, Belgium), magnification 1000x.

### *O*. *fruticosum* R1 progeny

An R1 generation was obtained from cross-breeding *O*. *fruticosum* control genotypes with selected Ri lines. Parent Ri lines were selected based on fertility characteristics and knowledge on the presence of pRi T-DNA genes and their copy number. An overview of the crosses and obtained seed populations is presented in S1 Table. Seed parent flowers were emasculated by removing the disc florets, and ray florets were pollinated once the stigma’s were visible. After pollination, each capitulum was sealed in a fine mesh bag to prevent from pollen contamination. Seeds were collected 2 to 3 months after pollination, imbibed in distilled water for 24 h and sown in a non-fertilized peat-sand (2:1 ratio) mixture. After 3 weeks, seedlings were transplanted to individual pots (Ø 5 cm) and grown as described above. In total, 40 R1 plants were maintained in the greenhouse. Leaf samples from these plants were collected for qPCR detection of the different pRi T-DNA genes. In addition, the *its* gene was used to verify DNA quality. The method for DNA extraction and qPCR conditions are as described in Desmet et al. [10]. Positive pRi progeny plants of Reg9 were also analyzed for CNV of the pRi1724 *rolA* gene using dPCR (as described above).

### Statistical analysis and software used

Chromosome and signal analysis was done using the DRAWID software version 0.26 [32]. All calculations of chromosome parameters and analyses were conducted in R (version 3.6.0) using the RStudio GUI (version 1.1.463). Analysis of variance (at significance level *P* < 0.05) was performed for both chromosome length and centromere index of the *O*. *fruticosum* karyotype via the ‘emmeans’ package in R (version 3.6.0). Post-hoc testing was done via Tukey’s HSD using the ‘cld’ function of the ‘multcomp’ package in R. Graphical idiogram construction was performed using R and Inkscape version 0.92.

## Results

### Presence of pRi T-DNA genes and analysis of copy number variation

Different combinations of pRi T-DNA are present in the *O*. *fruticosum* Ri lines. Reg1–8 carry both pRiA4 T_L_- and T_R_-DNA genes whereas Reg10 only harbors the T_L_-DNA genes. In Reg9 all four *rol*-genes are present. Reg11 also carries T_L_- and T_R_-DNA genes but does not contain the *rolA* gene. The copy number of the individual pRi T-DNA genes of the Ri lines was analyzed using dPCR with *rolA* as a proxy for all 4 *rol* genes (Table 2). Reg1–7 have a *rolA* copy number of at least 15. Exact numerical quantification was however not possible since high copy numbers hamper a more specific quantification. Analysis of the *aux* genes and *rolB*_*TR*_ showed lower copy numbers ranging from 3 to 7 copies. Reg8 is also characterized by high *rol* gene copy numbers (> 10), but has a single copy of the T_R_-DNA genes (Table 2). Reg9 carries 7 copies of the pRi1724 type *rol* genes. Reg10 harbors 3 copies of the pRiA4 *rol* genes. Interestingly, Reg11 has a copy number of 1 for the present *rol* and T_R_*-genes*.

**Table 2:** Copy number analysis of pRi T-DNA genes in *O*. *fruticosum* Ri lines. Values represent mean copy number ± SD (n = 2)(- = gene not present in this Ri line, NT = not tested, NA = gene not present in pRi used).

Ri line	*rolA*	*rolB*	*rolC*	*rolD*	*aux1*	aux2	*rolB*_*TR*_
Reg1	> 15	NT	NT	NT	7.0 ± 0.4	9.2 ± 0.2	6.5 ± 0.1
Reg2	> 15	NT	NT	NT	7.0 ± 0.4	9.0 ± 0.6	6.8 ± 0.3
Reg3	> 15	NT	NT	NT	7.4 ± 0.3	9.3 ± 0.2	7.1 ± 0.1
Reg4	> 15	NT	NT	NT	7.0 ± 0.0	9.6 ± 0.0	7.1 ± 0.3
Reg5	> 15	NT	NT	NT	6.5 ± 0.1	7.7 ± 0.7	5.5 ± 0.0
Reg6	> 15	NT	NT	NT	3.0 ± 0.0	3.0 ± 0.1	2.7 ± 0.1
Reg7	> 15	NT	NT	NT	6.9 ± 0.1	9.6 ± 0.1	6.2 ± 0.6
Reg8	> 10	NT	NT	NT	1.0 ± 0.1	1.0 ± 0.0	0.9 ± 0.1
Reg9	6.7 ± 0.0	6.5 ± 0.3	7.0 ± 0.3	7.2 ± 0.0	NA	NA	NA
Reg10	2.9 ± 0.0	3.0 ± 0.0	2.5 ± 0.2	2.9 ± 0.1	-	-	-
Reg11	-	1.0 ± 0.1	0.9 ± 0.0	1.0 ± 0.0	1.0 ± 0.1	1.1 ± 0.0	1.1 ± 0.0

### Karyotype analysis of *O*. *fruticosum*

**Fig 2:**
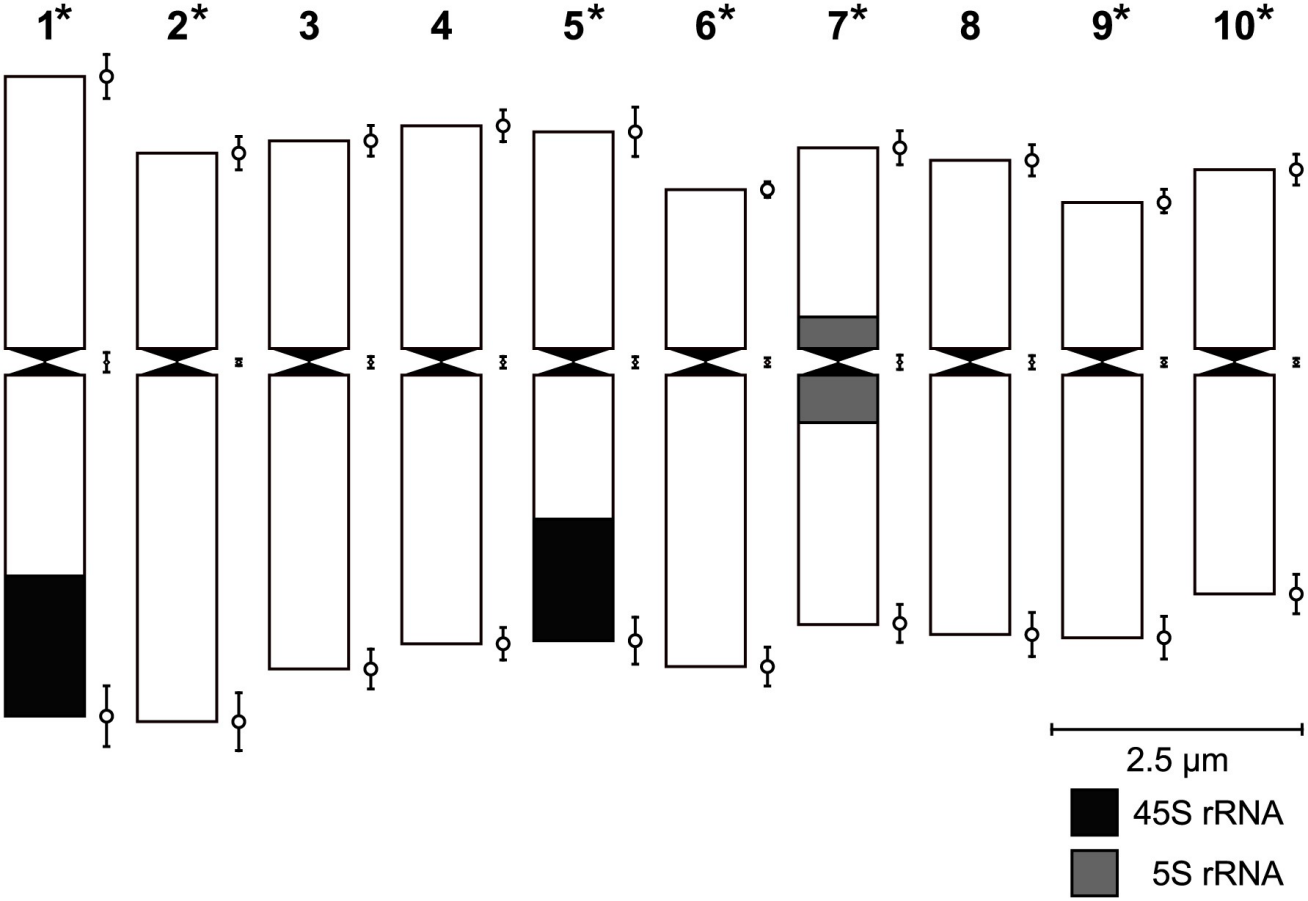
Monoploid idiogram of *O*. *fruticosum* (2n = 20, x = 10) chromosomes with signal localization of 45S and 5S rRNA. Error bars indicate the standard deviation for the short arm length, centromere position and long arm length at the top, middle and bottom left per chromosome, respectively.

**Fig 3:**
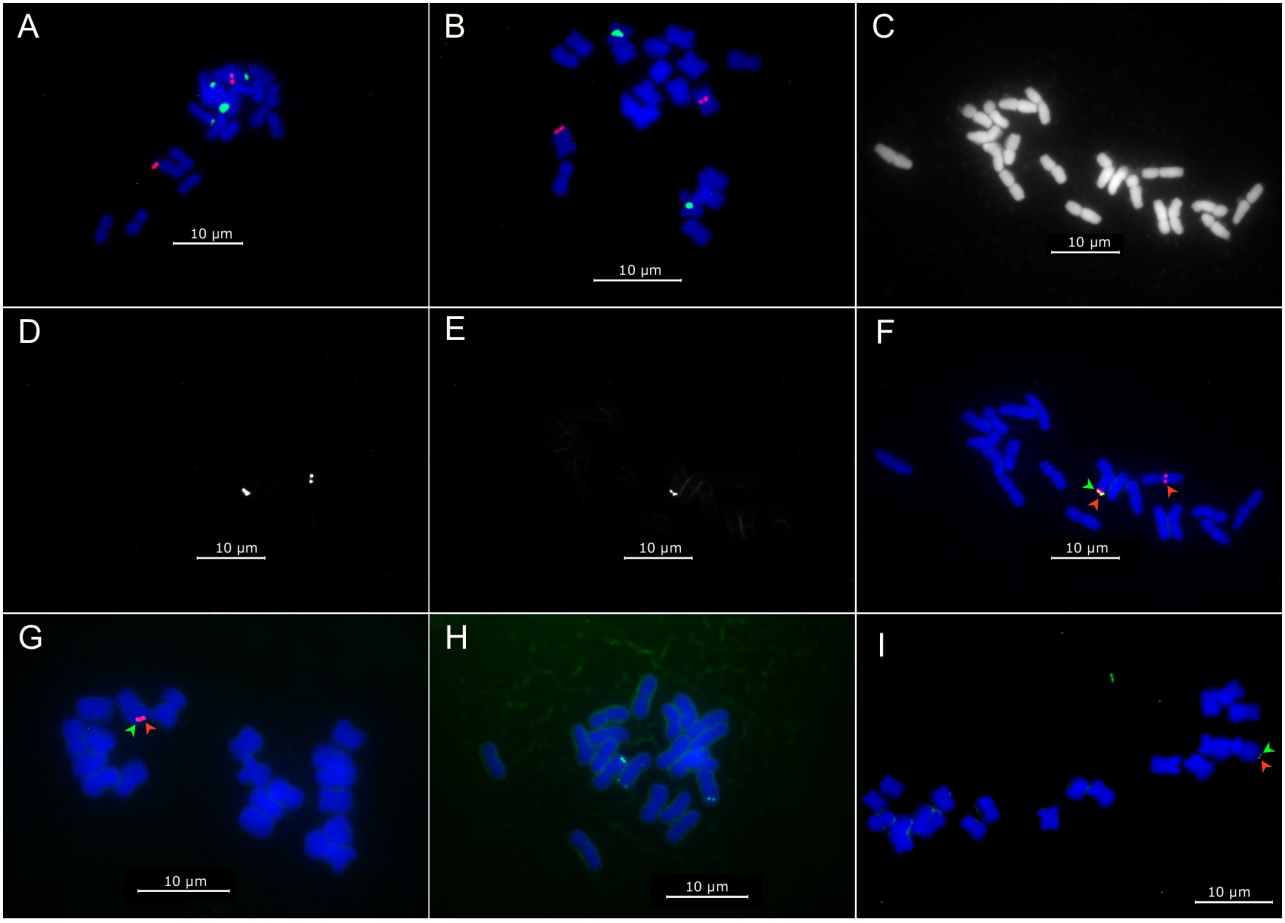
FISH mapping of pRi T-DNA, 45S and 5S rRNA on chromosomes of *O*. *fruticosum* Ri lines. (A) Multicolor FISH with pRiA4_ T_L_ (red) and pTa71_45S (green) probes on Reg3. (B) Multicolor FISH with pRiA4_ T_L_ (red) and pScT7_5S (green) probes on Reg3. Panels C, D, E and F display the same Reg3 metaphase with different probe overlays: (C) DAPI stained metaphase chromosomes of Reg3. (D) FISH localization of *rol* genes with the pRiA4_ T_L_ probe on Reg3. (E) FISH localization of *aux* genes with the pRiA4_T_R_ probe on Reg3. (F) Multicolor FISH overlay of C, D and E with pRiA4_ T_L_ (red) and pRiA4_T_R_ (green) probes on Reg3. (G) Multicolor FISH with pRiA4_ T_L_ (red) and pRiA4_T_R_ (green) probes on Reg8. (H) FISH with the pRi1724_*rol* (green) probe on Reg9. (I) Multicolor FISH with pRiA4_ T_L_ (green) and pRiA4_T_R_ (red) probes on Reg11. (Scale bar = 10 μm).

The somatic chromosome number of *O*. *fruticosum* of 2x = 2x = 20 was consistent among control genotypes and Ri lines. Significant differences in centromere index were observed and varied between 33.57 and 45.30 (Table 3). The majority of homologs are metacentric, except for the chromosomes 2, 6 and 9 which are submetacentric: 7M + 3sM (Fig 2). Chromosome length differed significantly between chromosomes and ranged from 3.99 to 6.11 μm (Table 3). FISH with the 45S rRNA probe showed strong long arm telocentric signals on chromosome 1 and on a chromosome of the 3, 4 or 5 group, allowing clear discrimination of at least one of these 3 chromosomes (Fig 3A). When 5S rRNA was used for FISH, a centromeric signal on the homologs of the chromosome 7 or 8 group was observed (Fig 3B). Based on chromosome morphology and FISH using 45S and 5S rRNA, 7 of the 10 homologs are readily identifiable (Fig 2).

**Table 3:** Chromosome characterization of *O*. *fruticosum*. Values represent mean ± SD and are calculated based on ‘n’ independent chromosome measurements, nomenclature according to Levan et al. [33]. Different letters within a column represent significant differences at *P* < 0.05 by Tukey’s HSD test.

Chromosome number	Chromosome length (μm)	Short arm length (μm)	Long arm length (μm)	Centromere index	Nomenclature	n
1	6.11 ± 0.98 a	2.71 ± 0.46	3.40 ± 0.63	44.64 ± 3.65 c	Metacentric	36
2	5.40 ± 0.94 ab	1.95 ± 0.36	3.45 ± 0.60	36.10 ± 1.73 e	Submetacentric	32
3	5.01 ± 0.69 bc	2.08 ± 0.33	2.93 ± 0.42	41.46 ± 2.85 cde	Metacentric	43
4	4.91 ± 0.63 bcd	2.23 ± 0.34	2.68 ± 0.35	45.30 ± 2.92 c	Metacentric	43
5	4.82 ± 0.97 cd	2.17 ± 0.52	2.65 ± 0.49	44.79 ± 2.90 c	Metacentric	36
6	4.51 ± 0.54 de	1.60 ± 0.18	2.91 ± 0.41	35.57 ± 2.56 de	Submetacentric	43
7	4.48 ± 0.66 cde	2.01 ± 0.36	2.49 ± 0.40	44.75 ± 3.80 c	Metacentric	42
8	4.48 ± 0.73 de	1.89 ± 0.34	2.59 ± 0.46	42.17 ± 3.60 cd	Metacentric	43
9	4.09 ± 0.64 e	1.47 ± 0.23	2.62 ± 0.45	35.98 ± 2.73 de	Submetacentric	42
10	3.99 ± 0.71 e	1.79 ± 0.33	2.19 ± 0.42	45.03 ± 2.68 c	Metacentric	45

### FISH mapping of T_L_- and T_R_-DNA in Ri lines

**Fig 4:**
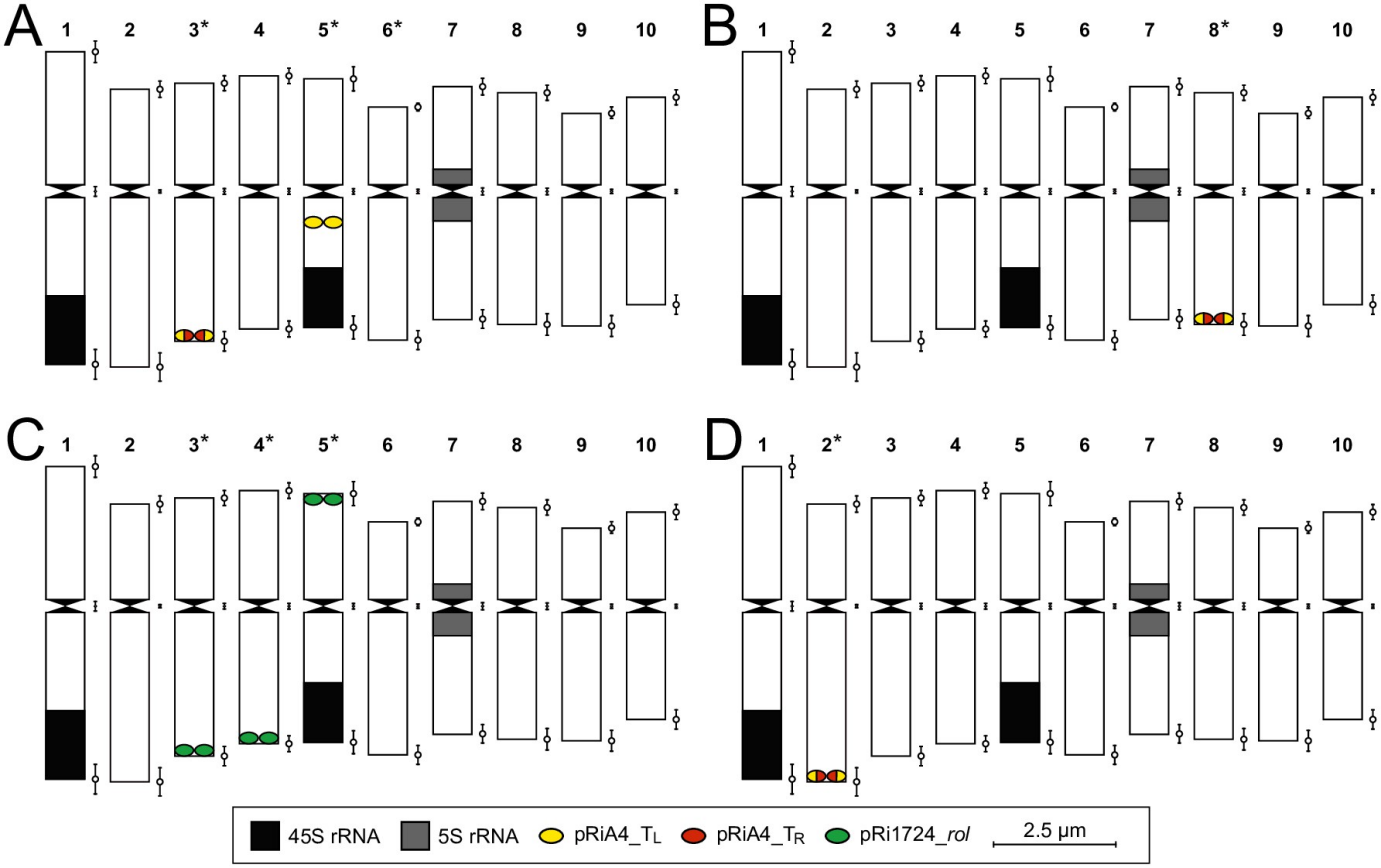
Monoploid idiogram of *O*. *fruticosum* Ri lines Reg3 (A), Reg8 (B), Reg9 (C) and Reg11 (D). Chromosomes are marked with signal localization tags of the 45S rRNA, 5S rRNA and pRi specific T-DNA probes (* T-DNA probe signals are located on one of the homologous chromosomes).

FISH experiments on the different Ri lines using pRi T-DNA, 45S and 5S rRNA probes showed varying signal numbers and signal intensity. In Reg3, the pRiA4_T_L_ probe resulted in 2 clear signals putatively located on a homologue of chromosome 3 and 5 (Fig 3C, 3D and 3F). The location of the signals differed; one signal was located near the telomere-end of the long arm of chromosome 3, the second signal was towards the centromeric region of the long arm of chromosome 5 (Fig 4A). The pRiA4_T_R_ probe showed 1 signal which co-localized with the pRiA4_T_L_ signal on a chromosome 3 homologue (Fig 3C, 3E and 3F). FISH using the same probes in Reg8 resulted in a single, co-localized long arm telocentric signal for both the pRiA4_T_L_ and the pRiA4_T_R_ probes, presumably located on a chromosome 8 homologue (Fig 4B). However, the intensity of the pRiA4_T_R_ signal was substantially lower compared to pRiA4_T_L_, suggesting a potential difference in copy number (Fig 3G). For localization of pRi1724 T-DNA in Reg9, a specific pRi1724_*rol* probe was used that resulted in 3 unique signals (Fig 3H). One signal was located on the telocentric part of the short arm of the 45S rRNA harboring chromosome of the 3, 4 or 5 group (Fig 4C). The other 2 signals are located on the remaining chromosomes of the 3, 4 or 5 group, both on the telocentric end of the long arm (Fig 4C). FISH with the pRiA4_T_L_ and the pRiA4_T_R_ probes conducted with Reg11 showed one low intensity co-localized telocentric signal located on chromosome 2 (Figs 3I and 4D).

### pRi T-DNA and copy number segregation in R1 progeny

The R1 generation consisted of 40 plants, originating from 11 populations (i.e. crosses, S1 Table). From these, 27 had inherited T-DNA genes (S2 Table). Progeny obtained from T_L_- and T_R_-DNA carrying Ri lines displayed separate segregation of the T_L_-DNA in plants R1_4 and R1_21. In all other pRiA4 progeny plants inherited both T_L_- and T_R_-DNA. Progenies with only the pRiA4 T_R_-DNA were not found. A *rol*^+^:*rol*^-^ segregation ratio of 16:4 was obtained for Reg9 derived R1 progenies, confirming that Reg9 harbors multiple pRi1724 *rol* gene loci. However, statistical assessment of segregation ratios was not possible due to the relative low numbers of seeds obtained per cross. Additional copy number analysis of the pRi1724 *rolA* gene of these plants revealed copy number segregation into 2, 3, 4 and 5 copies (Table 4). This indicates that the original Reg9 lines contains 7 copies distributed over 3 separate loci.

**Table 4:** Copy number analysis of *rolA* in *rol* positive *O*. *fruticosum* R1 progeny of Reg9. Values represent mean copy number ± SD (n = 2).

Population	Plant ID	Copy number
o2 x Reg9	R1_18	4.3 ± 0.6
Reg9 x o2 (I)	R1_23	3.2 ± 0.1
Reg9 x o2 (I)	R1_24	3.4 ± 0.4
Reg9 x o2 (I)	R1_25	3.5 ± 0.2
Reg9 x o2 (I)	R1_27	3.7 ± 0.0
Reg9 x o2 (I)	R1_28	4.8 ± 0.1
Reg9 x o2 (II)	R1_29	5.1 ± 0.1
Reg9 x o2 (II)	R1_31	1.6 ± 0.1
Reg9 x o2 (II)	R1_32	2.2 ± 0.6
Reg9 x o2 (II)	R1_33	4.0 ± 0.3
Reg9 x o2 (III)	R1_34	4.0 ± 0.5
Reg9 x o2 (III)	R1_35	5.2 ± 0.0
Reg9 x o2 (IV)	R1_36	3.6 ± 0.0
Reg9 x o2 (IV)	R1_37	1.9 ± 0.1
Reg9 x o2 (IV)	R1_38	1.8 ± 0.0
Reg9 x o2 (IV)	R1_39	5.0 ± 0.1

## Discussion

Ri lines derived from co-cultivation with wild type rhizogenic agrobacteria are characterized by specific phenotypic alterations that, together, make up the Ri phenotype [4]. This phenotype is generally characterized by a compact and bushy growth habit. Ri lines are often short, densely branched plants with reduced apical dominance and display increased rooting ability [7]. In *O*. *fruticosum*, Ri lines also display smaller flowers, shortened peduncles and, to a lesser extent, wrinkled leaves [10]. Since some of these traits are undesirable Ri lines are regarded as pre-breeding material due to the variety of phenotypic modifications [38–40]. Moreover, since both positive and less desirable traits occur simultaneously, Ri lines are often used for introgression breeding of specific traits, e.g. improved compactness [41, 42]. However, Ri lines have a distinct unique molecular composition which constitutes different degrees of the Ri phenotype [5, 43, 44]. To estimate the potential breeding value of *O*. *fruticosum* Ri lines, a protocol for the combined molecular and cytogenetic characterization was developed. In a previous work, qPCR analyses of Reg1 –Reg10 showed the presence of specific pRi T-DNA genes in these lines [10]. In eight of these lines, the integration of both T_L_- and T_R_-DNA had occurred, whereas in Reg10 only the T_L_-DNA was present. The *O*. *fruticosum* Ri phenotype generally consists of a compact plant habit characterized by shortened internodes, increased branching, decreased peduncle length and flower diameter. In our study however, the degree of compactness varied (1) from line to line, and (2) according to the *O*. *fruticosum* genotype from which the Ri line originated. For further breeding a careful selection of Ri lines is necessary. Ideally, the compact plant stature should be maintained, including the shortened peduncles, but decreased flower size should be avoided. As such, it is recommended to select those lines that offer maximal segregation of Ri traits [7], i.e. lines with higher copy numbers of the *rol*-genes located on different loci on the chromosomes.

It is generally accepted that transgene inheritance and segregation acts according to Mendelian principles [45]. For pRi T-DNA in specific, early breeding experiments conducted by Tepfer [4] showed the inheritance to R1 progeny, which coincided with the inheritance of two distinct transformed phenotypes in specific ratios, implying Mendelian segregation of pRi T-DNA. Segregation ratios are strongly affected by the copy number of the inserted transgenes [46]. In Cape daisy Ri lines, a variety of different copy numbers was observed, ranging from single inserts (Reg11), to intermediate (Reg9 and Reg10) and high copy numbers (> 15 copies, Reg1–8), irrespective of the *O*. *fruticosum* genotype or the bacterial strain used for transformation. Gelvin [19] states that integrated T-DNA copy numbers generally range from a single to tens of copies, with higher copy number being exceedingly rare. In contrast, the obtained copy numbers in this study were consistently high, often exceeding more than 10 estimated copies of the T-DNA. This is especially remarkable since Arqua1 is often described as a low virulence strain that yields low copy number transformants [47]. Guerche et al. [38] conducted CNV in *Brassica napus* Ri lines (based on Southern hybridization) and found copy numbers ranging from 1 to maximum 4. Similar lower copy numbers have been described in Ri lines of *Aesculus hippocastanum* [48], *Antirrhinum majus* [49], *Cichorium intybus* [50] and *Kalanchoe blossfeldiana* [11]. From literature at the moment of writing, the highest recorded copy number in Ri lines obtained with wild type *R*. *rhizogenes* is 7 [51]. These findings indicate a potential relationship between copy number distribution of Ri lines and the *R*. *rhizogenes* strain used.

Inheritance and segregation ratios are also strongly affected by the number of loci where T-DNA integration occurs. Transgene locus numbers have traditionally been determined by segregation analysis of the transformed phenotype or genotype [52]. Godo et al. [53] conclude that pRi T-DNA was inserted at one locus in a *Nierembergia scoparia* Ri line based on a 1:1 segregation ratio. Similarly, Handa [49] describes an *A*. *majus* Ri line with multiple T_L_-DNA integrations based on a 36 *rol*^+^: 4 *rol*^-^ segregation ratio. Relying on segregation ratio data alone to confirm the number of transgene loci is however unreliable as high numbers of progenies need to be analyzed for accurate assumptions. Moreover, as cross-breeding or selfing in *O*. *fruticosum* often yields relative low numbers of seedlings per cross (<10), obtaining accurate segregation ratios is very cumbersome. In addition, CNV does not offer helpful information in determining the number of T-DNA loci and hence the segregation potential. A more reliable method for characterization of transgene loci is through FISH [52]. FISH targeting the pRi T-DNA in *O*. *fruticosum* Ri lines revealed up to 3 independent integration loci. Interestingly, variation in T-DNA copy number and insertion loci was correlated with the plasmid structure of the *R*. *rhizogenes* strain. The number of insertion loci was higher in the MAFF210266 derived Ri line (Reg9) than in the Arqua1 (Reg3, Reg8) and ATCC15834 (Reg11) lines. To our knowledge, wild type pRi T-DNA locus characterization has previously only been performed by Ambros et al. [25], who report on two unique integration loci of pRi8196 T-DNA in hairy root lines of *Crepis capillaris*. Localization of tumor-inducing plasmid T-DNA (pTi T-DNA) in plants has been performed more often and usually one or two loci are detected [52, 54]. Although broadly speaking, agrobacteria have the propensity to deliver native T-DNA in low copy numbers and limited numbers of integration loci, a single FISH signal location can harbor multiple loci [55]. The minimum detectable distance between adjacent target sites is primarily determined by the spatial resolution of the specific detection method [56]. De Jong et al. [57] indicate that, though the resolution of mitotic metaphase chromosomes ranges from 2–5 Mbp, it is significantly affected by the level of chromatin condensation. Also, Harwood et al. [58] showed that FISH using mitotic metaphases is sufficiently sensitive to detect single T-DNA copies. We were also able to successfully detect a single, yet truncated, T_L_-DNA copy of approximately 3 kb on chromosome 2 of *O*. *fruticosum* Ri line Reg11. Single copy insertions such as T_L_-DNA in Reg11 and T_R_-DNA in Reg8 were however characterized by lower signal intensity. A similar relationship between copy number per insertion locus and signal intensity was also observed in potato [59].

Based on FISH and dPCR analysis, both the integration of full-size and truncated T_L_- and T_R_-DNA fragments was observed in the *O*. *fruticosum* Ri lines. Although complete T-DNA integration is often assumed, the detection of all T-DNA open reading frames (ORF) is only reported in two separate studies. Taneja et al. [60] investigated the presence of 23 pRiA4 ORFs in *Catharanthus roseus* hairy roots. Complete and truncated integration of the T_L_- and T_R_-DNA was observed, but the complete integration of ORF’s was more common. A similar result was obtained by Ray et al. [61] in *Rauvolfia serpentina* roots, however integration of T_R_-DNA ORF’s was more rare. Interestingly, in both these studies, the integration of pure T_R_-DNA ORF’s was not observed. The pRiA4 T-DNA has a split physical nature that allows for independent integration of the T_L_- and T_R_-DNA [1, 62]. By performing multi-color FISH, we were able to establish that the T_R_-DNA consistently co-localized with a T_L_-DNA signal. Insertion loci and patterns of separate T-DNA’s found on split type plasmids such as pRiA4 or pTiAch5 [63, 64] have however not yet been studied in great detail. Here, we have found a different integration pattern for the T_L_-DNA and T_R_-DNA in our *O*. *fruticosum* Ri lines. Whereas the T_L_-DNA segregates into progenies alone, the T_R_-DNA is always found to inherit together with T_L_-DNA. This is because of the fact that when the T_R_-DNA is present, it consistently co-localizes on a single locus with the T_L_-DNA. This is consistent with literature findings where plants that solely harbor the T_R_-DNA have never been described. Both Taylor et al. [65] and Guivarc’h et al. [66] suspected that transfer of only T_R_-DNA was a rare or species dependent occurrence, resulting in detrimental effects that hamper the regeneration of this specific genotype. In this study, the T_R_-DNA was only present in conjunction with T_L_-DNA and no segregation of these T-DNAs was observed in the R1 progenies. As such, breeding for plants with only T_R_-DNA is probably not feasible with the current *O*. *fruticosum* Ri lines.

Characterization of transgene loci in a variety of plant species has shown that insertions can occur at many different chromosomes [20, 67–69]. The specific site of insertion has been linked to expression patterns of transgenes, indicating they occur preferentially into transcription units [70–72]. From a pathogenic point of view, the integration into the gene space of the host genome would ensure maximal chances of oncogene expression. However, the randomness behind T-DNA integration is heavily plant species dependent [73]. The majority of characterized integration loci had a telocentric location, which is in agreement with the reported tendency of integration at distal chromosome regions [52, 69, 74]. However, we found that definitively allocating the insertion loci to specific chromosomes is complicated by a lack of chromosome specific markers and morphological distinctiveness between them. As such, insights related to preferential insertion sites will require more detailed cytological evaluation. To this end the development of chromosome specific markers could assist in chromosome specific allocation of the T-DNA insert.

The combination of accurate CNV through dPCR and FISH mapping of T-DNA insertion loci will facilitate future screening and selection of Ri lines with unique genetic constitutions. Selection of lines that can yield different T-DNA copy numbers by segregation will allow the study of the relationship between copy number and Ri phenotype. The current study allows us to compare earlier described phenotype data [10] with the copy number of the T-DNA’s. Reg1–8 all harbor a high number of pRiA4 type T-DNA. Reg3 harbors T-DNA on two loci, whereas Reg8 has a single insertion locus. However, further optimization of the dPCR protocol will be required in order to accurately determine higher copy numbers in future [75]. Based on the CNV and FISH results, we hypothesized that segregation of pRi1724 T-DNA in Reg9 progeny was very likely to occur based on the 7 copies spread over 3 loci. Indeed, CNV performed on Reg9 progeny showed segregation from the original 7 copies to either 2, 3, 4 or 5 copies. Based on this segregation pattern, the integration loci of Reg9 presumably harbor 2, 2 and 3 copies of pRi1724 rol genes each.

Taken together, characterization of integration loci in 4 Ri lines showed that multiple copies can be integrated at a single or multiple loci. Based on FISH results of Ri lines that carry both T_L_- and T_R_-DNA, the integration of both T-DNA segments is not completely independent as FISH signals consistently co-localized at presumable the same locus. The results also indicate that the combined FISH and CNV approach is a viable method to predict copy number segregation in progeny populations. The detailed molecular and cytogenetic characterization of *O*. *fruticosum* Ri lines described in this study, work synergistically in predicting the segregation of pRi T-DNA loci in progenies. By establishing and optimizing protocols for CNV and FISH it is possible to screen a larger set of Ri lines with the aim of selecting optimal lines for further breeding. This selective approach will narrow down the potential pool of breeding parents and can thus effectively prevent the use of Ri lines that offer a low likelihood of copy number segregation. Doing so allows researchers and breeders to save precious time and resources in breeding programs. Copy number segregation and the stacking of multiple Ri loci in progenies is hypothesized to be the fasted method for introgression of Ri traits in existing genepools [7]. In terms of future perspectives, we hypothesize that combining the findings of this study with Ri lines and introgression breeding will provide an optimal and efficient avenue for creating both genetic diversity in many horticultural crops while simultaneously selecting for optimal compactness, effectively eliminating the need for chemical growth regulation. This integrated approach will be viable for a plethora of crops and will help advance the implementation of Ri technology at a broad scale.

## Supporting information

S1 Fig Restriction site map for BfaI and EcoRI in the pRi1724 T-DNA used for copy number analysis of T-DNA genes of *R*. *rhizogenes*. (TIFF)

S1 Table Overview of the R1 populations obtained from cross-breeding *O*. *fruticosum* control genotypes and Ri lines. Germination rate = germinated / total number of seeds obtained per cross. Each plant obtained from germinated seeds was given a unique plant ID.
(DOCX)

S2 Table qPCR detection of pRi T-DNA genes in Reg 11 and R1 progeny populations of *O*. *fruticosum*. A positive (strain used to obtain the Ri line) and negative control (original genotypes either used to obtain the Ri line or used as parent) per set of progenies tested in the same qPCR run was included. Values represent the quantification cycle (Cq) values per gene. Values of T-DNA genes in bold indicate positive amplification based on Cq and amplicon melting profile, NA = gene not present in pRi used.
(DOCX)

S1 File (DOCX)
