# Supplementary figures and images for: Molecular and cytogenetic characterization of Osteospermum fruticosum lines harboring wild type pRi rol genes
Source: PLoS One. 2024 Sep 19;19(9):e0306905. doi: 10.1371/journal.pone.0306905 (PMC11412668; doi:10.1371/journal.pone.0306905)

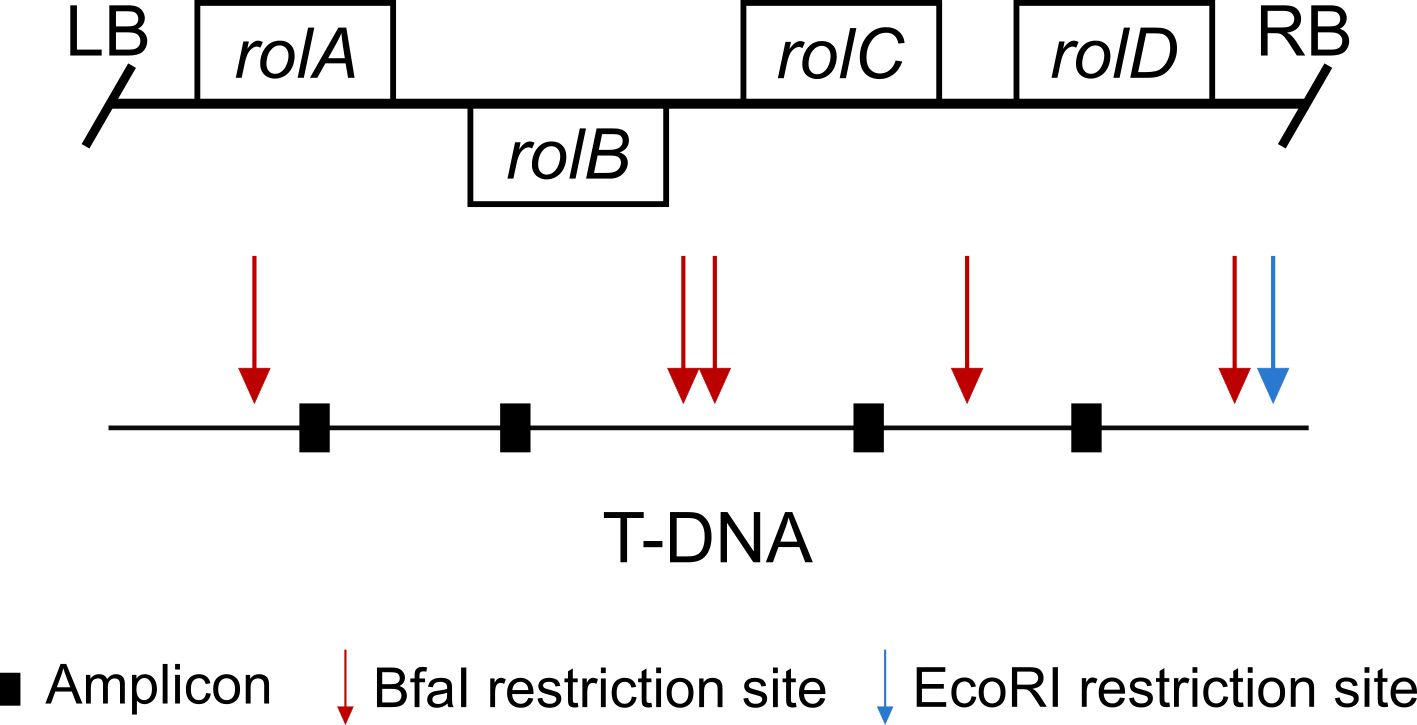

Supplement: S1 Fig — (TIFF) [file pone.0306905.s001.tiff]
