# Supplementary material for: Molecular and cytogenetic characterization of Osteospermum fruticosum lines harboring wild type pRi rol genes
Source: PLoS One. 2024 Sep 19;19(9):e0306905. doi: 10.1371/journal.pone.0306905 (PMC11412668; doi:10.1371/journal.pone.0306905)
Supplement: S1 Table — Germination rate = germinated / total number of seeds obtained per cross. Each plant obtained from germinated seeds was given a unique plant ID. (DOCX) [file pone.0306905.s002.docx]

**S2 Table** Overview of the R1 populations obtained from cross-breeding *O. fruticosum* control genotypes and Ri lines (germination rate: germinated / total number of seeds obtained per cross). Each plant obtained from germinated seeds was given a unique plant ID.

| Population | Germination rate | Plant ID |
| --- | --- | --- |
| o4 x Reg3 | 7 / 7 | R1_1 🡪 R1_7 |
| Reg10 x o2 (I) | 6 / 7 | R1_8 🡪 R1_13 |
| Reg10 x o2 (II) | 3 / 5 | R1_14 🡪 R1_16 |
| o2 x Reg9 | 2 / 8 | R1_17 🡪 R1_18 |
| Reg2 x o6 | 2 / 4 | R1_19 🡪 R1_20 |
| o4 x Reg6 | 1 / 1 | R1_21 |
| o3 x Reg5 | 1 / 1 | R1_22 |
| Reg9 x o2 (I) | 6 / 7 | R1_23 🡪 R1_28 |
| Reg9 x o2 (II) | 5 / 6 | R1_29 🡪 R1_33 |
| Reg9 x o2 (III) | 2 / 4 | R1_34 🡪 R1_35 |
| Reg9 x o2 (IV) | 5 / 7 | R1_36 🡪 R1_40 |
