# Supplementary material for: Molecular and cytogenetic characterization of Osteospermum fruticosum lines harboring wild type pRi rol genes
Source: PLoS One. 2024 Sep 19;19(9):e0306905. doi: 10.1371/journal.pone.0306905 (PMC11412668; doi:10.1371/journal.pone.0306905)
Supplement: S2 Table — A positive (strain used to obtain the Ri line) and negative control (original genotypes either used to obtain the Ri line or used as parent) per set of progenies tested in the same qPCR run was included. Values represent the quantification cycle (Cq) values per gene. Values of T-DNA genes in bold indicate positive amplification based on Cq and amplicon melting profile, NA = gene not present in pRi used. (DOCX) [file pone.0306905.s003.docx]

**S3 Table** qPCR detection of pRi T-DNA genes in Reg 11 and R1 progeny populations of *O. fruticosum*. A positive (strain used to obtain the Ri line) and negative control (original genotypes either used to obtain the Ri line or used as parent) per set of progenies tested in the same qPCR run was included. Values represent the quantification cycle (Cq) values per gene. Values of T-DNA genes in bold indicate positive amplification based on Cq and amplicon melting profile, NA = gene not present in pRi used.

| Population | Plant ID / sample | *rolA* | *rolB* | *rolC* | *rolD* | *its* | *aux1* | *aux2* | *rolB_TR_* |
| --- | --- | --- | --- | --- | --- | --- | --- | --- | --- |
| - | Reg11 | 35.0 | **22.0** | **21.3** | **21.5** | **12.2** | **21.2** | **20.8** | **22.1** |
| - | o1 | 33.4 | 40.0 | 33.1 | 27.0^a^ | 12.7 | 33.7 | 38.2 | 30.4 |
| - | ATCC15834 | 14.1 | 13.9 | 13.4 | 13.2 | 37.0 | 13.3 | 13.0 | 14.4 |
| o4 x Reg3 | R1_1 | **18.6** | **18.2** | **17.9** | **17.5** | 12.7 | **19.1** | **18.6** | **20.1** |
|  | R1_2 | 33.8 | 35.2 | 31.9 | 25.8^a^ | 12.9 | 30.5 | 30.2 | 30.1 |
|  | R1_3 | **18.5** | **18.6** | **18.1** | **18.1** | 12.6 | **19.5** | **18.9** | **20.3** |
|  | R1_4 | **19.0** | **19.0** | **18.7** | **18.0** | 12.0 | 31.8 | 32.2 | 30.1 |
|  | R1_5 | 33.1 | 33.0 | 30.3 | 26.0^a^ | 13.2 | 29.8 | 30.2 | 29.0 |
|  | R1_6 | 32.0 | 40.0 | 32.0 | 26.5^a^ | 13.7 | 29.0 | 29.1 | 28.5 |
|  | R1_7 | **19.7** | **19.6** | **19.0** | **19.3** | 13.7 | **19.7** | **19.0** | **20.6** |
| Reg10 x o2 (I) | R1_8 | 31.6 | 33.2 | 31.5 | 26.4^a^ | 12.3 | 30.0 | 31.3 | 29.5 |
|  | R1_9 | 31.9 | 33.6 | 29.8 | 26.2^a^ | 13.5 | 29.9 | 30.6 | 29.2 |
|  | R1_10 | 33.0 | 35.5 | 31.3 | 26.2^a^ | 13.0 | 31.8 | 31.8 | 29.5 |
|  | R1_11 | **20.9** | **20.9** | **20.3** | **20.3** | 13.6 | 30.6 | 30.7 | 29.1 |
|  | R1_12 | **20.9** | **20.8** | **19.9** | **20.1** | 12.8 | 29.9 | 29.5 | 28.7 |
|  | R1_13 | 33.7 | 37.0 | 33.7 | 28.1^a^ | 14.9 | 28.9 | 28.7 | 28.1 |
| Reg10 x o2 (II) | R1_14 | 32.8 | 34.7 | 32.0 | 26.8^a^ | 12.2 | 40.0 | 30.1 | 29.5 |
|  | R1_15 | 33.6 | 40.0 | 33.7 | 26.7^a^ | 12.8 | 30.1 | 30.2 | 28.9 |
|  | R1_16 | **21.4** | **21.2** | **20.5** | **20.2** | 12.5 | 28.1 | 27.6 | 28.3 |
| o4 x Reg6 | R1_21 | **23.0** | **23.1** | **22.2** | **22.4** | 13.6 | 32.5 | 31.5 | 30.2 |
| - | o2 | 33.5 | 36.3 | 31.8 | 25.9^a^ | 11.8 | 34.3 | 36.9 | 30.2 |
| - | o4 | 32.8 | 36.2 | 29.7 | 26.5^a^ | 12.6 | 32.2 | 36.6 | 31.0 |
| - | Arqua1 | 14.1 | 14.0 | 13.3 | 13.1 | 36.6 | 13.3 | 12.9 | 14.5 |
|  |  |  |  |  |  |  |  |  |  |
| o2 x Reg9 | R1_17 | 34.5 | 35.3 | 31.9 | 32.6 | 12.7 | NA | NA | NA |
|  | R1_18 | **19.7** | **19.9** | **19.4** | **20.7** | 12.1 | NA | NA | NA |
| Reg9 x o2 (I) | R1_23 | **20.8** | **20.7** | **20.2** | **21.6** | 13.4 | NA | NA | NA |
|  | R1_24 | **20.2** | **20.2** | **19.8** | **21.0** | 13.3 | NA | NA | NA |
|  | R1_25 | **20.7** | **20.9** | **20.3** | **21.5** | 13.5 | NA | NA | NA |
|  | R1_26 | 34.2 | 34.2 | 30.8 | 32.5 | 12.2 | NA | NA | NA |
|  | R1_27 | **19.6** | **19.6** | **19.1** | **20.5** | 12.3 | NA | NA | NA |
|  | R1_28 | **19.3** | **19.4** | **18.8** | **20.1** | 13.2 | NA | NA | NA |
| Reg9 x o2 (II) | R1_29 | **20.0** | **20.1** | **19.7** | **20.9** | 12.8 | NA | NA | NA |
|  | R1_30 | 32.7 | 33.4 | 32.1 | 32.6 | 13.5 | NA | NA | NA |
|  | R1_31 | **21.1** | **21.2** | **20.7** | **22.0** | 12.8 | NA | NA | NA |
|  | R1_32 | **20.1** | **20.2** | **19.9** | **21.0** | 13.2 | NA | NA | NA |
|  | R1_33 | **19.6** | **19.6** | **19.2** | **20.3** | 13.8 | NA | NA | NA |
| Reg9 x o2 (III) | R1_34 | **19.7** | **19.7** | **19.2** | **20.3** | 12.3 | NA | NA | NA |
|  | R1_35 | **19.8** | **19.8** | **19.4** | **20.6** | 13.1 | NA | NA | NA |
| Reg9 x o2 (IV) | R1_36 | **20.3** | **20.5** | - | **21.8** | 12.5 | NA | NA | NA |
|  | R1_37 | **22.0** | **22.1** | **21.6** | **22.9** | 13.9 | NA | NA | NA |
|  | R1_38 | **22.0** | **22.2** | **21.6** | **22.9** | 13.8 | NA | NA | NA |
|  | R1_39 | 32.6 | 33.4 | 31.9 | 32.6 | 12.4 | NA | NA | NA |
|  | R1_40 | **19.9** | **20.0** | **19.6** | **20.7** | 12.5 | NA | NA | NA |
| - | o2 | 37.2 | 35.7 | 31.2 | 31.8 | 11.8 | NA | NA | NA |
| - | o4 | 36.5 | 34.5 | 31.7 | 31.2 | 12.6 | NA | NA | NA |
| - | MAFF210266 | 16.6 | 16.6 | 16.1 | 17.2 | 36.7 | NA | NA | NA |
|  |  |  |  |  |  |  |  |  |  |
| Reg2 x o6 | R1_19 | **17.5** | **17.5** | **17.1** | **17.3** | 12.8 | **17.9** | **17.5** | **19.0** |
|  | R1_20 | **18.1** | **18.2** | **17.7** | **17.7** | 12.7 | **19.0** | **18.6** | **20.1** |
| o3 x Reg5 | R1_22 | **18.7** | **18.7** | **18.2** | **18.2** | 14.4 | **19.6** | **19.1** | **20.6** |
| - | o2 | 33.8 | 36.8 | 32.3 | 25.5 | 12.3 | 34.5 | 36.6 | 31.2 |
| - | o3 | 33.1 | 35.1 | 32.1 | 26.4 | 14.2 | 34.0 | 40.0 | 30.5 |
| - | o6 | 33.7 | 40.0 | 33.8 | 26.0 | 13.2 | 35.1 | 38.2 | 29.1 |
| - | Arqua1 | 15.0 | 14.9 | 14.3 | 14.0 | 35.9 | 14.2 | 13.8 | 15.4 |

^a^ Melting profile showed a non-specific amplicon
