# Supplementary material for: Molecular and cytogenetic characterization of Osteospermum fruticosum lines harboring wild type pRi rol genes
Source: PLoS One. 2024 Sep 19;19(9):e0306905. doi: 10.1371/journal.pone.0306905 (PMC11412668; doi:10.1371/journal.pone.0306905)
Supplement: S1 File — (DOCX) [file pone.0306905.s004.docx]

**Molecular and Cytogenetic Characterization of *Osteospermum fruticosum* Lines Harboring Wild Type pRi *rol* Genes**

**SUPPLEMENTARY INFORMATION**

*Siel Desmet^1,2^, Katrijn Van Laere^1^, Johan Van Huylenbroeck^1^, Danny Geelen^2^, Ellen De Keyser^1^, Emmy Dhooghe^1^

^1^ Plant Sciences Unit, Flanders Research Institute for Agriculture, Fisheries and Food (ILVO), Caritasstraat 39, 9090 Melle, Belgium

^2^ Department Plants and Crops, Faculty of Bioscience Engineering, Ghent University, Coupure Links 653, 9000 Ghent, Belgium

*Corresponding author: Siel.Desmet@ilvo.vlaanderen.be

**S1 Fig** Restriction site map for *BfaI* and *EcoRI* in the pRi1724 T-DNA used for copy number analysis of T-DNA genes of *R.* *rhizogenes*.


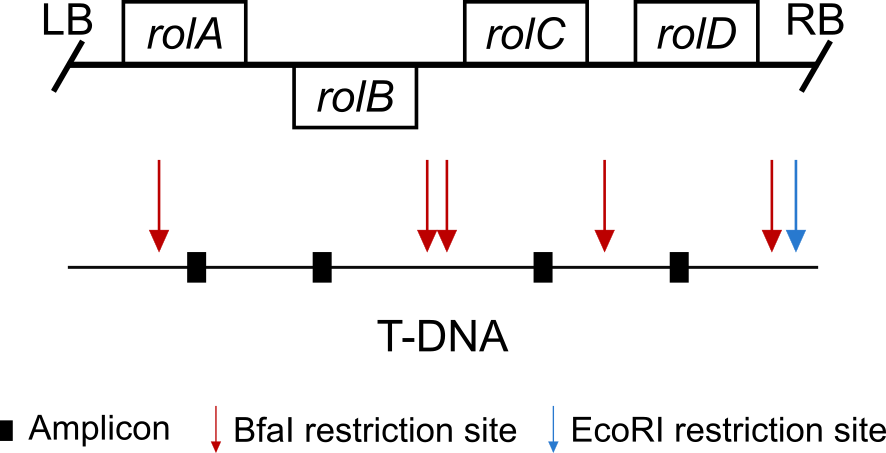


**S2 Table** Overview of the R1 populations obtained from cross-breeding *O. fruticosum* control genotypes and Ri lines (germination rate: germinated / total number of seeds obtained per cross). Each plant obtained from germinated seeds was given a unique plant ID.

| Population | Germination rate | Plant ID |
| --- | --- | --- |
| o4 x Reg3 | 7 / 7 | R1_1 🡪 R1_7 |
| Reg10 x o2 (I) | 6 / 7 | R1_8 🡪 R1_13 |
| Reg10 x o2 (II) | 3 / 5 | R1_14 🡪 R1_16 |
| o2 x Reg9 | 2 / 8 | R1_17 🡪 R1_18 |
| Reg2 x o6 | 2 / 4 | R1_19 🡪 R1_20 |
| o4 x Reg6 | 1 / 1 | R1_21 |
| o3 x Reg5 | 1 / 1 | R1_22 |
| Reg9 x o2 (I) | 6 / 7 | R1_23 🡪 R1_28 |
| Reg9 x o2 (II) | 5 / 6 | R1_29 🡪 R1_33 |
| Reg9 x o2 (III) | 2 / 4 | R1_34 🡪 R1_35 |
| Reg9 x o2 (IV) | 5 / 7 | R1_36 🡪 R1_40 |

**S3 Table** qPCR detection of pRi T-DNA genes in Reg 11 and R1 progeny populations of *O. fruticosum*. A positive (strain used to obtain the Ri line) and negative control (original genotypes either used to obtain the Ri line or used as parent) per set of progenies tested in the same qPCR run was included. Values represent the quantification cycle (Cq) values per gene. Values of T-DNA genes in bold indicate positive amplification based on Cq and amplicon melting profile, NA = gene not present in pRi used.

| Population | Plant ID / sample | *rolA* | *rolB* | *rolC* | *rolD* | *its* | *aux1* | *aux2* | *rolB_TR_* |
| --- | --- | --- | --- | --- | --- | --- | --- | --- | --- |
| - | Reg11 | 35.0 | **22.0** | **21.3** | **21.5** | **12.2** | **21.2** | **20.8** | **22.1** |
| - | o1 | 33.4 | 40.0 | 33.1 | 27.0^a^ | 12.7 | 33.7 | 38.2 | 30.4 |
| - | ATCC15834 | 14.1 | 13.9 | 13.4 | 13.2 | 37.0 | 13.3 | 13.0 | 14.4 |
| o4 x Reg3 | R1_1 | **18.6** | **18.2** | **17.9** | **17.5** | 12.7 | **19.1** | **18.6** | **20.1** |
|  | R1_2 | 33.8 | 35.2 | 31.9 | 25.8^a^ | 12.9 | 30.5 | 30.2 | 30.1 |
|  | R1_3 | **18.5** | **18.6** | **18.1** | **18.1** | 12.6 | **19.5** | **18.9** | **20.3** |
|  | R1_4 | **19.0** | **19.0** | **18.7** | **18.0** | 12.0 | 31.8 | 32.2 | 30.1 |
|  | R1_5 | 33.1 | 33.0 | 30.3 | 26.0^a^ | 13.2 | 29.8 | 30.2 | 29.0 |
|  | R1_6 | 32.0 | 40.0 | 32.0 | 26.5^a^ | 13.7 | 29.0 | 29.1 | 28.5 |
|  | R1_7 | **19.7** | **19.6** | **19.0** | **19.3** | 13.7 | **19.7** | **19.0** | **20.6** |
| Reg10 x o2 (I) | R1_8 | 31.6 | 33.2 | 31.5 | 26.4^a^ | 12.3 | 30.0 | 31.3 | 29.5 |
|  | R1_9 | 31.9 | 33.6 | 29.8 | 26.2^a^ | 13.5 | 29.9 | 30.6 | 29.2 |
|  | R1_10 | 33.0 | 35.5 | 31.3 | 26.2^a^ | 13.0 | 31.8 | 31.8 | 29.5 |
|  | R1_11 | **20.9** | **20.9** | **20.3** | **20.3** | 13.6 | 30.6 | 30.7 | 29.1 |
|  | R1_12 | **20.9** | **20.8** | **19.9** | **20.1** | 12.8 | 29.9 | 29.5 | 28.7 |
|  | R1_13 | 33.7 | 37.0 | 33.7 | 28.1^a^ | 14.9 | 28.9 | 28.7 | 28.1 |
| Reg10 x o2 (II) | R1_14 | 32.8 | 34.7 | 32.0 | 26.8^a^ | 12.2 | 40.0 | 30.1 | 29.5 |
|  | R1_15 | 33.6 | 40.0 | 33.7 | 26.7^a^ | 12.8 | 30.1 | 30.2 | 28.9 |
|  | R1_16 | **21.4** | **21.2** | **20.5** | **20.2** | 12.5 | 28.1 | 27.6 | 28.3 |
| o4 x Reg6 | R1_21 | **23.0** | **23.1** | **22.2** | **22.4** | 13.6 | 32.5 | 31.5 | 30.2 |
| - | o2 | 33.5 | 36.3 | 31.8 | 25.9^a^ | 11.8 | 34.3 | 36.9 | 30.2 |
| - | o4 | 32.8 | 36.2 | 29.7 | 26.5^a^ | 12.6 | 32.2 | 36.6 | 31.0 |
| - | Arqua1 | 14.1 | 14.0 | 13.3 | 13.1 | 36.6 | 13.3 | 12.9 | 14.5 |
|  |  |  |  |  |  |  |  |  |  |
| o2 x Reg9 | R1_17 | 34.5 | 35.3 | 31.9 | 32.6 | 12.7 | NA | NA | NA |
|  | R1_18 | **19.7** | **19.9** | **19.4** | **20.7** | 12.1 | NA | NA | NA |
| Reg9 x o2 (I) | R1_23 | **20.8** | **20.7** | **20.2** | **21.6** | 13.4 | NA | NA | NA |
|  | R1_24 | **20.2** | **20.2** | **19.8** | **21.0** | 13.3 | NA | NA | NA |
|  | R1_25 | **20.7** | **20.9** | **20.3** | **21.5** | 13.5 | NA | NA | NA |
|  | R1_26 | 34.2 | 34.2 | 30.8 | 32.5 | 12.2 | NA | NA | NA |
|  | R1_27 | **19.6** | **19.6** | **19.1** | **20.5** | 12.3 | NA | NA | NA |
|  | R1_28 | **19.3** | **19.4** | **18.8** | **20.1** | 13.2 | NA | NA | NA |
| Reg9 x o2 (II) | R1_29 | **20.0** | **20.1** | **19.7** | **20.9** | 12.8 | NA | NA | NA |
|  | R1_30 | 32.7 | 33.4 | 32.1 | 32.6 | 13.5 | NA | NA | NA |
|  | R1_31 | **21.1** | **21.2** | **20.7** | **22.0** | 12.8 | NA | NA | NA |
|  | R1_32 | **20.1** | **20.2** | **19.9** | **21.0** | 13.2 | NA | NA | NA |
|  | R1_33 | **19.6** | **19.6** | **19.2** | **20.3** | 13.8 | NA | NA | NA |
| Reg9 x o2 (III) | R1_34 | **19.7** | **19.7** | **19.2** | **20.3** | 12.3 | NA | NA | NA |
|  | R1_35 | **19.8** | **19.8** | **19.4** | **20.6** | 13.1 | NA | NA | NA |
| Reg9 x o2 (IV) | R1_36 | **20.3** | **20.5** | - | **21.8** | 12.5 | NA | NA | NA |
|  | R1_37 | **22.0** | **22.1** | **21.6** | **22.9** | 13.9 | NA | NA | NA |
|  | R1_38 | **22.0** | **22.2** | **21.6** | **22.9** | 13.8 | NA | NA | NA |
|  | R1_39 | 32.6 | 33.4 | 31.9 | 32.6 | 12.4 | NA | NA | NA |
|  | R1_40 | **19.9** | **20.0** | **19.6** | **20.7** | 12.5 | NA | NA | NA |
| - | o2 | 37.2 | 35.7 | 31.2 | 31.8 | 11.8 | NA | NA | NA |
| - | o4 | 36.5 | 34.5 | 31.7 | 31.2 | 12.6 | NA | NA | NA |
| - | MAFF210266 | 16.6 | 16.6 | 16.1 | 17.2 | 36.7 | NA | NA | NA |
|  |  |  |  |  |  |  |  |  |  |
| Reg2 x o6 | R1_19 | **17.5** | **17.5** | **17.1** | **17.3** | 12.8 | **17.9** | **17.5** | **19.0** |
|  | R1_20 | **18.1** | **18.2** | **17.7** | **17.7** | 12.7 | **19.0** | **18.6** | **20.1** |
| o3 x Reg5 | R1_22 | **18.7** | **18.7** | **18.2** | **18.2** | 14.4 | **19.6** | **19.1** | **20.6** |
| - | o2 | 33.8 | 36.8 | 32.3 | 25.5 | 12.3 | 34.5 | 36.6 | 31.2 |
| - | o3 | 33.1 | 35.1 | 32.1 | 26.4 | 14.2 | 34.0 | 40.0 | 30.5 |
| - | o6 | 33.7 | 40.0 | 33.8 | 26.0 | 13.2 | 35.1 | 38.2 | 29.1 |
| - | Arqua1 | 15.0 | 14.9 | 14.3 | 14.0 | 35.9 | 14.2 | 13.8 | 15.4 |

^a^ Melting profile showed a non-specific amplicon
